# Feasibility Study on Reusing Recycled Premixed Multi-Material Powder in the Laser Powder Bed Fusion Process for Thermal Management Application

**DOI:** 10.3390/mi16101186

**Published:** 2025-10-20

**Authors:** Shiming Gao, Shuo Qu, Junhao Ding, Haoming Mo, Xu Song

**Affiliations:** 1 Zhejiang Engineering Research Center of Advanced Water Conservancy Equipment, Zhejiang University of Water Resources and Electric Power, Hangzhou 310018, China; shiminggaozju@gmail.com; 2 Key Laboratory of Key Technologies for Mechanical Industry Hydroelectric Power Generation Pump Turbine, Zhejiang University of Water Resources and Electric Power, Hangzhou 310018, China; 3 Department of Mechanical and Automation Engineering, The Chinese University of Hong Kong, Shatin, Hong Kong SAR, China; jhding@link.cuhk.edu.hk (J.D.); hmmok@link.cuhk.edu.hk (H.M.)

**Keywords:** Cu-Ni, reused powder, laser powder bed fusion, heat exchanger

## Abstract

Large-scale applications of multi-material manufacturing technology face many challenges. One major issue is how to reuse the mixed powder left after printing. In this study, we propose using an effective structure design to compensate for the performance loss of reused materials, thereby achieving the purpose of reusing premixed waste powder in certain non-critical thermal management applications. Taking Cu and Ni premixture powder as an example, some explorations were then conducted on the feasibility of the proposed concept. The morphological inspection confirms that the powder mixture exhibits satisfactory homogeneity, while the Hall flow rate measurements reveal that its flowability is closer to that of pure Ni. The compression tests show that the fabricated Cu-Ni specimens have good energy absorption, whereas tensile tests reveal their favorable ductility. The numerical analysis indicates that the effect of convection heat transfer is much greater than that of conduction heat transfer. Heat transfer experiments show that the Cu-Ni heat exchanger exhibits comparable performance to pure Cu, with a heat transfer effectiveness deviation of less than 1.3%. Previous results indicate that effective structure design can offset the loss of material properties, allowing premixed powders to be utilized in heat exchanger production as a means of recycling waste powders.

## 1. Introduction

Laser powder bed fusion (LPBF), as one of the additive manufacturing technologies, has been widely used in aerospace [1,2], automotive [3,4], medical [5,6], and nuclear [7,8] fields, as well as other fields. It has the ability to process all metallic materials, including stainless steel [9,10], titanium alloy [11,12], Ni alloy [13,14], aluminum alloy [15,16], etc. Contrary to conventional subtractive techniques, in LPBF, a thin layer of powder is first spread over the building area using a scraper or roller; then, the laser beam selectively melts the particles according to the slice path. The final part is fabricated through this repeating layer-by-layer process. The nature of this technology indicates that it requires large amounts of powder to operate the machine, while only a small amount of powder is ultimately used to form the part. It has been reported that the cost of powder accounts for 10–50% of the cost of the manufactured part for most alloys [17]. Therefore, reducing manufacturing costs and protecting the environment have always been challenges facing LPBF technology.

Powder recycling and separation technology, as an effective approach to reducing production costs, has gained prominence alongside the advancement of LPBF technology. Given that LPBF is a complex process involving melting, solidification, and alloying, the characteristics of the powder (e.g., composition and morphology) may undergo changes after printing. Therefore, a series of studies was conducted on possible performance changes in recycled powders and their impact on the product. Jelis et al. [18] fabricated some 4340 steel specimens from virgin and recycled powders and studied their influence on mechanical properties. High oxygen content was detected in the recycled powder, and the tensile strength showed a downward trend with the increase in oxygen content. Gruber et al. [19] studied the effect of recycled 718 alloy powder on the fracture behavior of specimens. After 14 recycling cycles, a high concentration of Al-rich oxide particulates was observed in the printed parts, with damaged defects showing a clear correlation to powder degradation. Tradowsky et al. [20] investigated the impact of powder recycling on the mechanical properties of samples fabricated from AlSi10Mg powder. Their findings revealed that tensile properties deteriorated due to voids formed by the oxide film. Delacroix et al. [21] found that the oxygen content and ferrite phase fraction in 316L powder increased after powder recycling. Ahmed et al. [22] investigated the impact of powder recycling on the roughness, porosity, and tensile properties of fabricated 17-4PH parts. Compared to those made with virgin powder, samples printed using recycled powder showed a 54% increase in pore size, a 17% increase in top surface roughness, and a 7% reduction in ductility. The above-mentioned studies show that powder recycling in the LPBF process may change the characteristics of the powder and affect the performance of the product to a certain extent. In some critical applications, changes in powder properties can be a fatal problem. For example, in medical implants and aerospace sectors, maintaining high powder purity is critical to meeting safety standards and stringent performance. Therefore, exploring ways to reuse these substandard powders is urgent.

Multi-material manufacturing has recently attracted considerable attention due to its ability to precisely tailor localized properties (e.g., thermal and corrosion resistance) within components. Wei et al. [23] produced 316L/IN718 and 316L/Cu10Sn samples through a point-by-point delivery system. In their experiment, a sandwich layer distribution with good metallurgical bonding was obtained. Wits et al. [24] fabricated some functionally graded structures using IN718 and 316L powders and studied element diffusion in the transition zone. Through liquid state mixing and diffusion, a graduated region with homogeneous element distribution was observed in their results. Qu et al. [25] fabricated a full-composition Cu-Ni gradient alloy through in situ alloying, creating pure Cu and Ni powder in a single printing process. A gradient yield strength (231 to 445 MPa), tensile strength (303 to 488 MPa), and electrical conductivity (3.6% to 96% IACS) were achieved in their fabricated samples. Wen et al. [26] printed a CoCrMo/IN718 compositional graded alloy by developing a new spreading strategy. In experiments, they successfully achieved controlled changes in microstructure, hardness, and tensile properties. However, the large-scale application of the above-mentioned multi-material manufacturing technologies remains challenging. One of the difficulties is how to effectively reuse the remaining premixed powders after printing, thereby reducing manufacturing costs and protecting the environment.

Unfortunately, existing powder recycling and separation technologies are inadequate for the recycling of multi-material powders. Chivel et al. [27] proposed a method for separating multi-material powders based on particle size and effectively achieved powder screening. However, their study merely stated that no analogs were found and did not provide a detailed purity analysis. Furthermore, this method has certain requirements for the particle size differences of powders. Ullrich et al. [28] utilized centrifugal force to achieve multi-material powder separation. The premixed powder was placed in a centrifuge, and the density difference between the materials was used for separation and purification. A small portion of multi-component powder was still observed after screening. Binder et al. [29] adopted the magnetic properties of the powder to separate premixed CuCrZr and X3NiCoMoTi18-9-5 powders. Before separation, a series of complex inspections was required to determine its feasibility, including material properties (e.g., reflection, chemical stability, and plasticity) and powder characteristics (e.g., particle shape, particle size, and particle mass). This method, however, can only separate two premixed metal powders with obvious magnetic and non-magnetic differences. The above studies indicate that the separation and recycling of multi-material powders still face great challenges, especially for those premixed powders with similar properties. Considering the limitations of existing technologies and the fact that the expected properties of premixed powder products may be between those of the original powders, it is worth considering whether we can bypass this difficulty and instead use an effective structure design to compensate for the loss in powder performance, so as to achieve the reuse of these premixed powders in non-critical thermal management applications.

Triply periodic minimal surfaces (TPMSs), as a well-known porous structure, have been widely adopted to improve heat transfer performance in heat exchanger (HE) applications. A series of studies has been conducted to investigate its heat transfer mechanism and performance improvement effects. Huang et al. [30] successfully fabricated an aero-engine regenerator by employing Diamond and Fischer–Koch S lattice structures. Their experimental results demonstrated a remarkable enhancement in thermal performance, with the proposed structures achieving 37–86% improvement in heat transfer efficiency when compared to conventional printed circuit heat exchangers (PCHEs). Yan et al. [31] discovered that in a Re range of 200–500, the performance evaluation coefficient (PEC) of Diamond, Gyroid, and IWP was 90~110% higher than that of PCHE. A “merge–split” flow characteristic with several secondary circulations enhanced its heat transfer. Based on extensive simulation analysis, Qian et al. [32] optimized the conventional Gyroid and Diamond structures and designed two innovative TPMS HEs. Their experiment results indicated that the PEC values of Gyroid and Diamond improved by 200% and 170%, respectively. By developing a comprehensive simulation model, Peng et al. [33] found that the Diamond-structured heat exchanger (HE) demonstrated a remarkable 7.5-fold enhancement in heat transfer rate compared to conventional plate heat exchangers. This significant improvement was primarily attributed to the unique eccentric helical flow patterns generated within the intersecting channels. Wang et al. [34] analyzed the heat transfer mechanisms of IWP, Nerovious, Fischer–Koch S, and Primitive TPMS structures. Their findings demonstrated that periodic flow acceleration/deceleration, secondary flows, and frequency changes in flow direction played a key role in enhancing heat transfer. The above-mentioned studies showed that the TPMS structure can greatly improve heat transfer performance, and its enhanced method mainly relies on convection. The authors’ own previous work [35] also found that there are a large number of spiral motions in the channel, and these spiral motions increase rapidly as the cell size decreases. Therefore, for these TPMS heat exchangers, whether the thermal conductivity of the material is still important to its overall heat transfer performance is a question worth considering.

Inspired by this, in this study, we firstly propose to utilize the advantages of the TPMS structure itself to compensate for the loss of material performance and consider its application in heat exchanger design. By applying HEs made of premixed powder to replace HEs made of virgin powder, we hope to realize the reuse of premixed powder in the heat transfer field. To verify the feasibility of this idea, we take Cu and Ni premixed powder as an example, which are powders leftover from the manufacturing process of in situ-alloyed Cu-Ni compositional graded alloy [25]. Corresponding extensive investigations were systematically carried out to validate the feasibility of the previously proposed concept. The powder’s characteristics were first inspected to confirm its manufacturability. Then, the microstructure and surface morphology were examined to determine part quality. Next, the compression and tensile tests were conducted to evaluate its mechanical properties. Furthermore, a numerical analysis was performed to reveal the flow characteristics. Finally, the produced Cu-Ni heat exchanger was compared with the pure Cu heat exchanger to evaluate the difference in heat transfer performance. The results of this work are expected to provide some useful guidance for the reuse of Cu and Ni premixed powders and extend to other materials.

## 2. Design and Experimental Details

### 2.1. TPMS Heat Exchanger Design

In this study, a Diamond-type triply periodic minimal surface (TPMS) structure is chosen as the research object. Its mathematical representation can be expressed through the following implicit function [36]:

(1) $$F\left(x,y,z\right)=\sin\left(x\right)\cdot \sin\left(y\right)\cdot \sin\left(z\right)+\sin\left(x\right)\cdot \cos\left(y\right)\cdot \cos\left(z\right)+\cos\left(x\right)\cdot \sin\left(y\right)\;\cdot \cos\left(z\right)+\cos\left(x\right)\cdot \cos\left(y\right)\cdot \sin\left(z\right)\;+\;c$$

Here, the variable c is the offset distance of the isosurface, which controls the partition wall thickness; x, y, and z are Cartesian coordinates.

**Figure 1:**
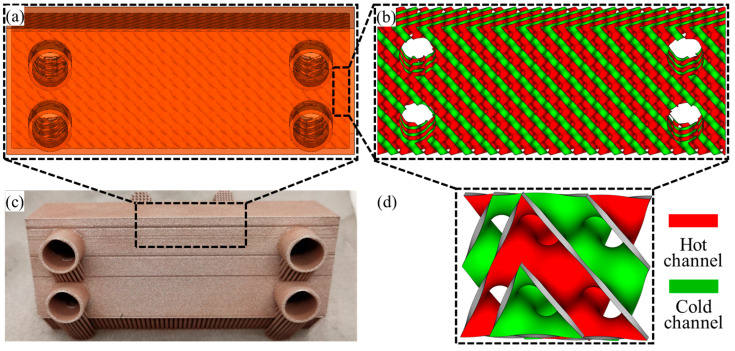
Schematic of designed TPMS heat exchanger: (**a**) overall HE shapes; (**b**) TPMS heat exchange core; (**c**) fabricated Cu-Ni alloy HE; (**d**) TPMS unit cell.

To ensure good formability and prevent leakage, the geometric parameters of the designed TPMS heat exchangers are carefully optimized as follows: unit cell size of 5 mm, wall thickness of 300 μm, and overall core dimensions of 70 mm (L) × 25 mm (W) × 15 mm (H). The fluid flow channels were designed with 7.4 mm diameter inlet/outlet ports and 8 mm connection lengths. The whole model is generated through our self-developed MATLAB code (Version: R2022a). The detailed design and fabrication of the parts are shown in Figure 1.

For microstructure and mechanical property analysis, three dog-bone specimens with a gauge dimension of 15 mm (L) × 4 mm (W) × 1.5 mm (H) are utilized to calculate their tensile strength. The samples for the compression test are 4 × 4 × 4 cell arrays. A cube with dimensions of 2 mm× 2 mm ×2 mm is utilized to inspect the microstructure.

### 2.2. Laser Powder Bed Fusion

Test samples and heat exchangers are fabricated by a high-precision LPBF machine (Han’s Laser M100μ). The raw material is a mixture of Cu and Ni powder, which is the remaining powder after the in situ alloying of a Cu-Ni composition gradient alloy. The powder has a spherical morphology, and its particle size is in the range of 5 μm~25 μm. Before powder spreading, ball milling is conducted at 200 RPM for two hours to improve powder uniformity. The additive manufacturing parameters are optimized as follows: laser power is 100 W, scanning speed is 600 mm/s, layer thickness is 10 μm, and hatch distance is 50 μm. To enhance structural integrity and minimize anisotropic effects, a 67° interlayer rotation strategy was implemented between successive layers. The substrate adopts a 316L stainless steel plate, and the whole deposition process is conducted in a controlled Nitrogen atmosphere, maintaining oxygen and H_2_O concentrations below 500 ppm.

### 2.3. Heat Transfer Evaluation

Heat transfer performance is evaluated by the following indicators:

The average heat transfer rate is calculated by [36,37]

(2) $${\dot{Q}}_{avg}=\frac{{\dot{m}}_{h}\cdot c_{p,h}\cdot \left(T_{h,in}-T_{h,out}\right)+{\dot{m}}_{c}\cdot c_{p,c}\cdot \left(T_{c,in}-T_{c,out}\right)}{2}$$

Here, the subscripts *h* and *c* present the hot and cold sides of the heat exchange medium; $\dot{m}$ is the mass flow rate; and $c_{p}$ is the specific heat capacity.

The total heat transfer coefficient, commonly referred to as the overall heat transfer coefficient (U), can be mathematically expressed as follows:

(3) $$U=\frac{{\dot{Q}}_{avg}}{A\cdot {∆T}_{lmtd}}$$

where A is the total heat exchange area in HE, and $∆T_{lmtd}$ is the logarithmic mean temperature difference, calculated as

(4) $$∆T_{lmtd}=\frac{∆T_{1}-∆T_{2}}{ln\frac{∆T_{1}}{∆T_{2}}}$$

where $∆T_{1}=T_{h,in}-T_{c,out}$; $∆T_{2}=T_{h,out}-T_{c,in}$.

The heat transfer effectiveness, *ε*, is determined using the following expression [36,37]:

(5) $$\varepsilon =\frac{{\dot{Q}}_{avg}}{{\dot{Q}}_{max}}$$

where ${\dot{Q}}_{max}$ is the maximum heat transfer rate, which is equal to ${\dot{C}}_{min}\left(T_{h,in}-T_{c,in}\right)$; ${\dot{C}}_{min}$ is the minimum heat capacity of the cold and hot fluids.

### 2.4. Characterization

The microstructure was observed via RH-2000 optical microscopy. Before the investigation, samples were first polished with metallographic sandpaper and then finely polished with 0.5 μm diamond solution. A solvent of 5 g FeCL_3_ + 85 mL alcohol + 15 mL HCl was utilized to etch the samples. The etching time was 15 s. The phase composition was analyzed by a JSM 6400 scanning electron microscope with energy-dispersive X-ray spectroscopy (EDS) detectors. The compression performance was evaluated by an Alliance RT/50 compressive machine at a rate of 0.2 mm/min. The tensile strength was measured by the Tinius Olsen H5KS tensile machine at a rate of 0.96 mm/min.

### 2.5. Experimental and Numerical Procedures

The heat transfer experiments were conducted according to references [38,39]. The volumetric flow rates were precisely regulated within the range of 30–90 L/h. The inlet temperatures of cold and hot water were set to 25 °C and 50 °C, respectively. The temperature variation was measured by PT100 thermocouples, while the pressure drop was detected through a differential pressure transmitter. To minimize heat loss and ensure measurement accuracy, the heat exchanger assembly was comprehensively insulated with thermal insulation cotton throughout the experimental procedure.

**Figure 2:**
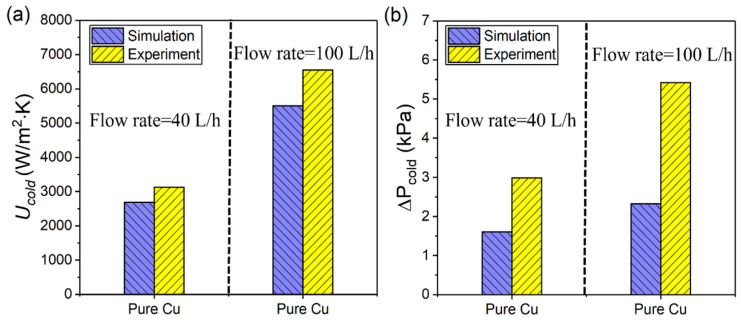
Simulated and experimental heat transfer performance: (**a**) overall heat transfer coefficient ($U_{cold}$); (**b**) pressure drop in cold channel (${∆P}_{cold}$).

The numerical analysis was performed based on a three-dimensional model identical to the actual object. The Ansys Fluent software 2021R2 was used to solve the conjugate heat transfer and steady-state Reynolds–Navier–Stokes equations. Based on previous studies [30,32,40,41], the fluid heat transfer within the TPMS structures predominantly occurred in the turbulent regime; hence, a shear stress transport (SST) k-ω model was employed to capture the turbulence effect. The inlet boundary conditions adopted a velocity inlet, and the outlet boundary condition adopted a pressure outlet. Before the numerical analysis, mesh independence was checked to ensure simulation accuracy. A pure Cu heat exchanger was chosen as a representative case. Table 1 presents the computed cold/hot outlet temperatures versus the grid number for an inlet flow rate of 100 L/h with *T_cold_* = 25 °C and *T_hot_* = 50 °C. Considering the trade-off between accuracy and computational cost, a grid system consisting of 50.91 million polyhedral elements was ultimately selected for the entire model. The simulation reliability was validated by a corresponding experiment, as shown in Figure 2. The deviation in the overall heat transfer coefficient ($U_{cold}$) reached 15.89% at a flow rate of 100 L/h. The pressure drop (${∆P}_{cold}$) exhibited a deviation of 57.10%, which can be primarily attributed to surface roughness and geometric errors that were not considered by the simulation model. However, the model still had certain reliability in analyzing heat transfer characteristics.

## 3. Results and Discussion

### 3.1. Microstructure

**Figure 3:**
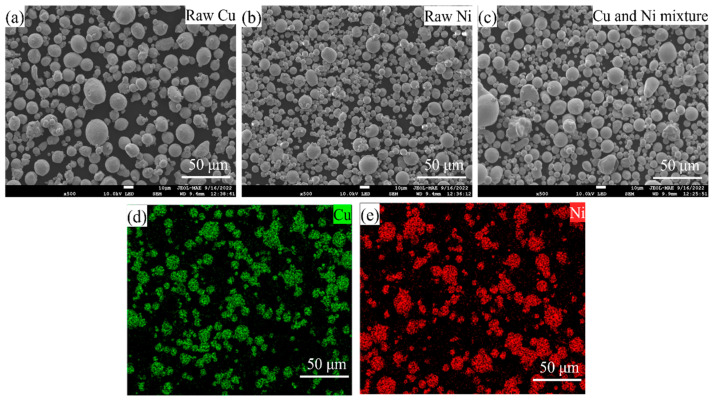
Powder characteristics of (**a**) virgin Cu powder; (**b**) virgin Ni powder; (**c**) reused Cu and Ni mixture; (**d**) Cu powder distribution in mixture; and (**e**) Ni powder distribution in mixture.

**Figure 4:**
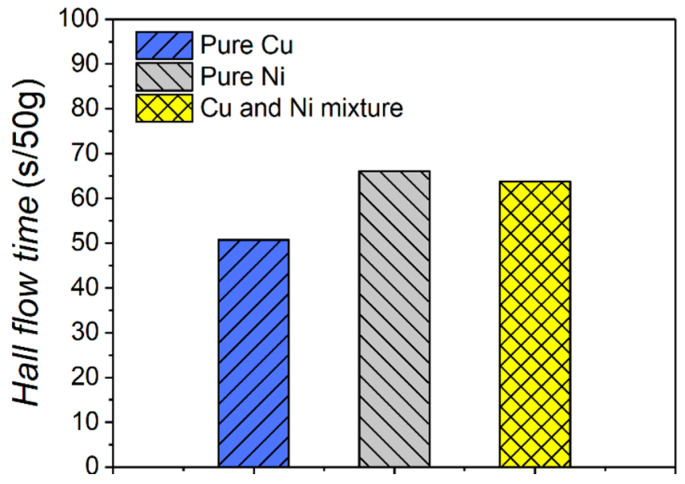
Hall flow time for 50 g materials.

The characteristics of virgin powder and the mixture of Cu and Ni waste powder are shown in Figure 3. The morphology of virgin Cu powder presents larger spherical particles with less satellite powder. In contrast, the morphology of virgin Ni powder shows smaller spherical particles with a great deal of satellite powder. This indicates that the flowability of pure Ni powder may be worse than that of pure Cu powder. Interestingly, without a screening operation, the morphology of the Cu and Ni mixture exhibits medium-sized spherical particles with a certain number of satellites, and no obvious morphology deterioration was observed after printing. The Hall flow rate measurements indicate that pure Cu exhibits superior flowability compared to pure Ni, while the Cu and Ni mixture demonstrates intermediate behavior between the two, with a tendency closer to that of pure Ni, as illustrated in Figure 4. The element distributions in the powder mixture are shown in Figure 3d,e. The EDS results show that the distribution of Cu and Ni in the mixed powder is relatively uniform, and the total ratio of Cu:Ni is about 1:0.93. The powder mixture has acceptable homogeneity for final product manufacturing.

**Figure 5:**
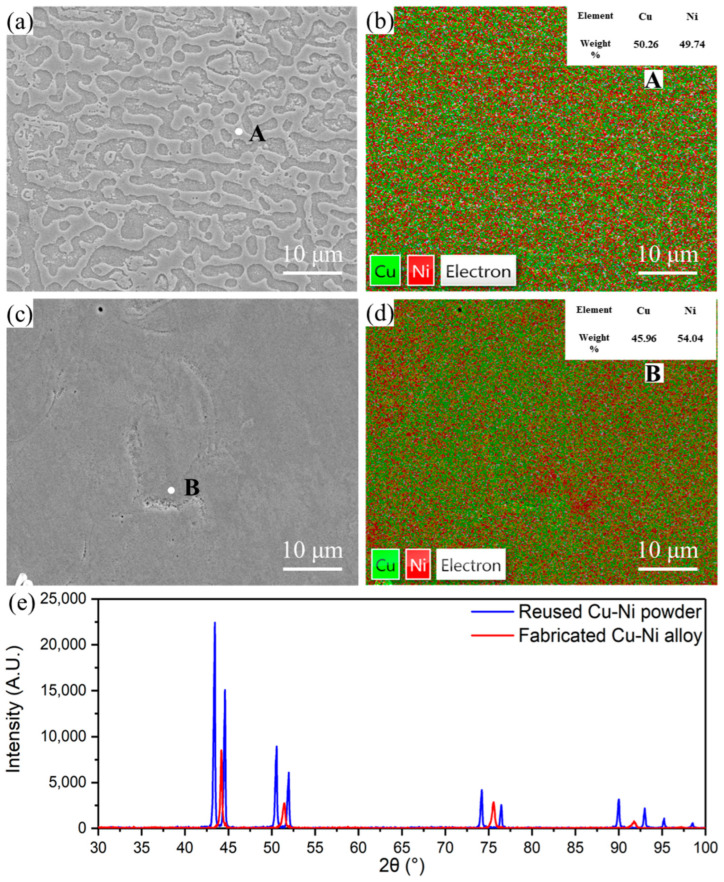
Microstructure and composition analysis of fabricated Cu-Ni alloy after etching: (**a**) solid solution microstructure; (**b**) distribution of Cu and Ni element in (**a**); (**c**) incomplete solid solution microstructure; (**d**) distribution of Cu and Ni element in (**c**); (**e**) XRD pattern of reused Cu and Ni mixed powder and fabricated Cu-Ni alloy.

The microstructures of the fabricated Cu-Ni alloy specimens are shown in Figure 5. The corroded surfaces appear in two cases. One is the complete Cu-Ni solid solution, as shown in Figure 5a. Similar results were also reported in references [42,43]. The EDS surface scanning indicates that these areas have relatively uniform element distribution, as shown in Figure 5b. The point analysis shows that the ratio of Cu:Ni is close to 1:1. Another is the incomplete Cu-Ni solid solution, as shown in Figure 5c, where the dendrite phase cannot be clearly distinguished due to the poor corrosion effect. The EDS surface scanning shows that there is a certain degree of inhomogeneity in the distribution of the Cu and Ni elements in these areas, as shown in Figure 5d. These results indicate that local inhomogeneities in the composition and texture of the final specimens exist due to unalloyed powder. However, intermetallic compounds are not observed due to the good mutual solubility of Cu and Ni, as shown in Figure 5e. Similar results are reported in [25].

**Figure 6:**
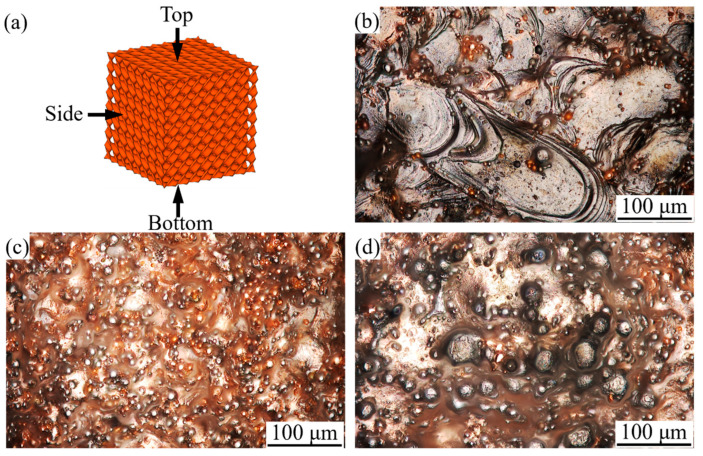
Surface morphology at different positions of fabricated specimens: (**a**) 4 × 4 × 4 Diamond model; (**b**) top surface (Ra: 6.47 μm); (**c**) side surface (Ra:11.48 μm); (**d**) bottom surface (Ra:21.11 μm).

The surface morphology of fabricated 4 × 4 × 4 Diamond cell arrays is shown in Figure 6. The top surface is relatively smooth, and the scan path is visible, while the side and bottom surfaces are uneven, and several powder particles have adhered. The measured arithmetic mean roughness (Ra) values of the top, side, and bottom surfaces are 6.47 μm, 11.48 μm, and 21.11 μm, respectively. This phenomenon is attributed to powder adhesion. Due to laser melting, the top surface is almost free of powder, but the side and bottom surfaces are surrounded by the powder bed. As the temperature drops below the solidification point, the unmelted powder at the edge of the molten pool will be stuck, causing a rough surface. The bottom surface has a larger surface roughness value than the sides due to the additional staircase effect, as described in reference [44].

### 3.2. Mechanical Properties

**Figure 7:**
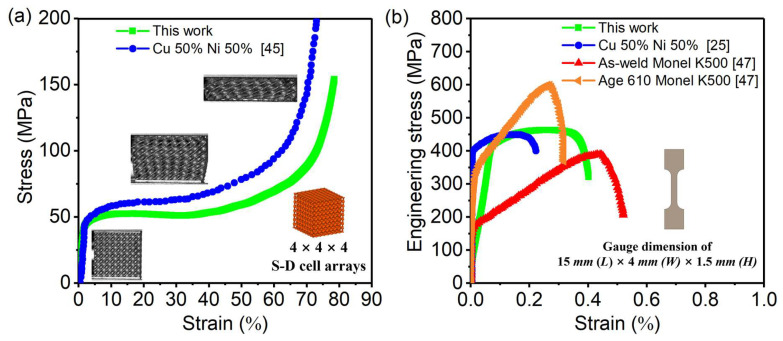
Mechanical properties of fabricated Cu-Ni specimens: (**a**) compression; (**b**) tensile.

The compression stress–strain curves of fabricated 4 × 4 × 4 Diamond cell arrays are shown in Figure 7a. The curves mainly present three distinguished stages. In the initial stage, the TPMS structure shows a nearly linear elastic property. Then, as the compression progresses further, a long stress plateau appears. At this stage, the value of stress almost remains constant, while the strain gradually increases to about 32%, which is very suitable for energy absorption. Afterward, the structure enters the densification stage. Here, the stress increases rapidly until the TPMS structure is fully densified. This rapid rise in stress is mainly caused by the self-contact of TPMS shell lattices. Compared to the compression strain curve of a Cu_0.5_Ni_0.5_ TPMS reported in the literature [45], our curve exhibits slightly inferior performance, primarily due to structural differences. The TPMS in the literature has a unit size of 2 mm, whereas our unit size is 5 mm. Smaller unit sizes generally result in better mechanical properties, as described in [46].

The tensile stress–strain curves of fabricated dog-bone specimens are shown in Figure 7b. The yield strength (YS) and ultimate tensile strength (UTS) are about 415 MPa and 465 MPa, respectively. The elongation reaches about 0.37~0.4. This mechanical property shows better ductility than the Cu_0.5_Ni_0.5_ curve reported in the literature [25] and is much better than the as-weld Monel K500 (Cu_0.317_Ni_0.683_) curve described in reference [47]. The Age 610 Monel K500 curve has the highest UTS because it has higher Ni content, and the aging treatment can significantly improve the uniformity of the structure. The compression and tensile stress–strain curves show that the performance of the Cu-Ni alloy made of powder mixture is close to that of existing alloys.

**Figure 8:**
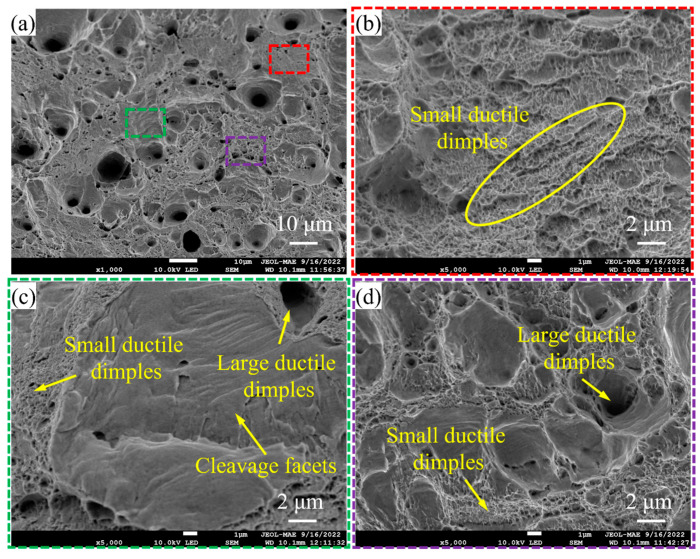
SEM photos of (**a**) fracture surfaces of tensile specimens; (**b**) magnified view of the red area in (**a**); (**c**) magnified view of the green area in (**a**); (**d**) magnified view of the purple area in (**a**).

The fracture surfaces of the tensile specimens are shown in Figure 8. No obvious voids or unmelted powder are found in the fractography, which indicates the adopted laser energy density is suitable for Cu and Ni powder mixture fabrication. The magnified views in Figure 8b–d show that there are several dimple-like features and a few cleavage facets on the fracture section. Since the proportion of small and large dimples in the cross-section is much larger than that of the cleavage facets, the fracture mode for fabricated Cu-Ni alloy specimens almost presents a ductile mode corresponding to Figure 7b. Similar results were also found in the literature [47].

### 3.3. Heat Transfer Characteristics

**Figure 9:**
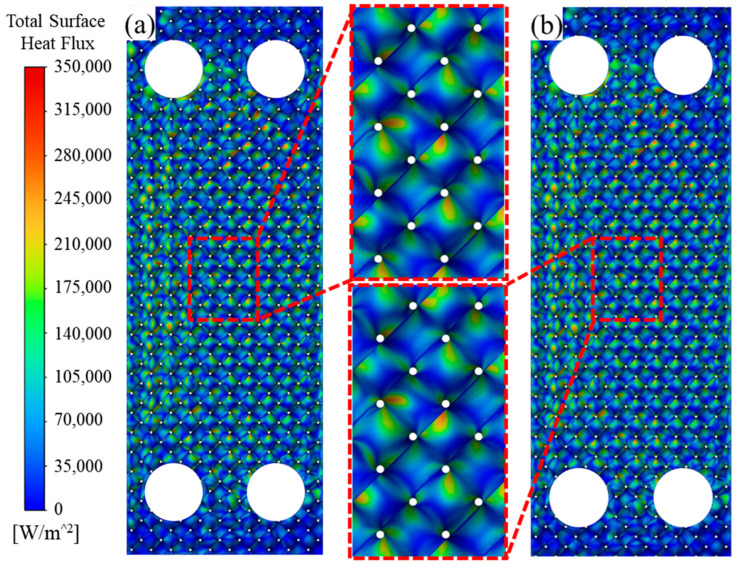
Simulated total surface heat flux distribution in proposed HE: (**a**) pure Cu; (**b**) Cu-Ni alloy.

The thermal conductivity of Cu-Ni alloy is very sensitive to its composition. Ackerman et al. [48] reported that when the Cu content changes from 99.5% to 97%, the thermal conductivity of Cu-Ni alloy decreases from 304 W/(m·K) to 141 W/(m·K). To verify whether the advantages of the TPMS structure itself can make up for the loss of material conductivity, numerical simulations are first performed to analyze its heat transfer characteristics. Figure 9a shows the total surface heat flux of the designed pure Cu heat exchanger under the following simulation conditions: inlet flow rate = 100 L/h, *T_cold_* = 25 °C, and *T_hot_* = 50 °C. It is found that the total surface heat flux presents a certain eccentric distribution. The left side has a larger value than the right side due to the distribution of the water inlets. The stronger turbulence causes this phenomenon. Interestingly, the Cu-Ni heat exchanger shows almost the same total surface heat flux distribution as the Cu heat exchanger, as shown in Figure 9b. This indicates that the effect of convection heat transfer is much greater than conduction heat transfer, thereby weakening the impact of material differences. The uneven distribution of heat flux on the node surface is caused by the eccentric flow formed by the continuous reversal of the water in the TPMS channels.

**Figure 10:**
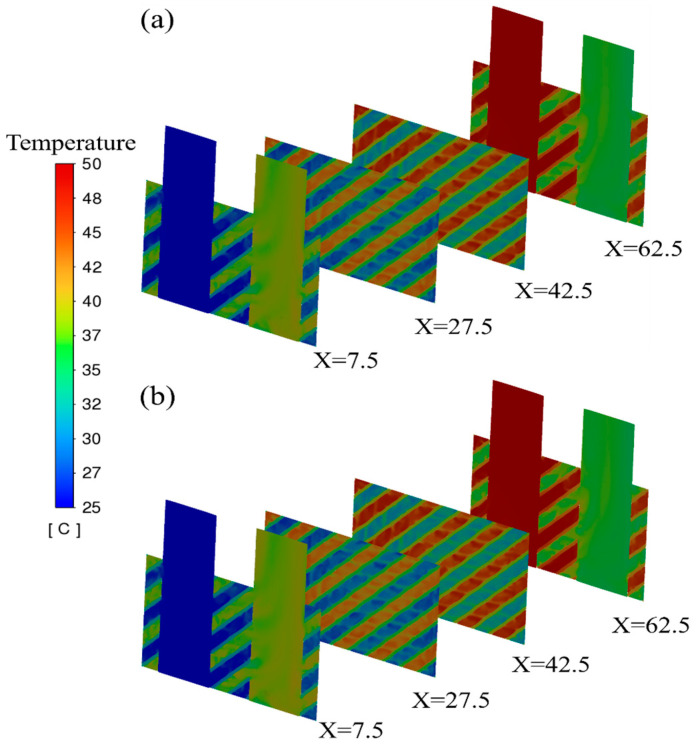
Temperature distribution of proposed HEs: (**a**) pure Cu; (**b**) Cu-Ni alloy.

Figure 10 shows the temperature distribution of the proposed pure Cu and Cu-Ni alloy HEs at lengths of X = 7.5 mm, 27.5 mm, 42.5 mm, and 62.5 mm. The similar temperature field means that there is almost no difference in the heat transfer performance between these two heat exchangers. The wavy temperature distribution on the planes of X = 27.5 mm and 42.5 mm is highly related to its flow characteristics. When the water flows through the intertwined channels, the fluid inside will be squeezed and produce high-speed shear with the surface, resulting in localized areas of the wall having high heat transfer coefficients. The material thermal conductivity then becomes less important.

### 3.4. HE Performance Evaluation

**Figure 11:**
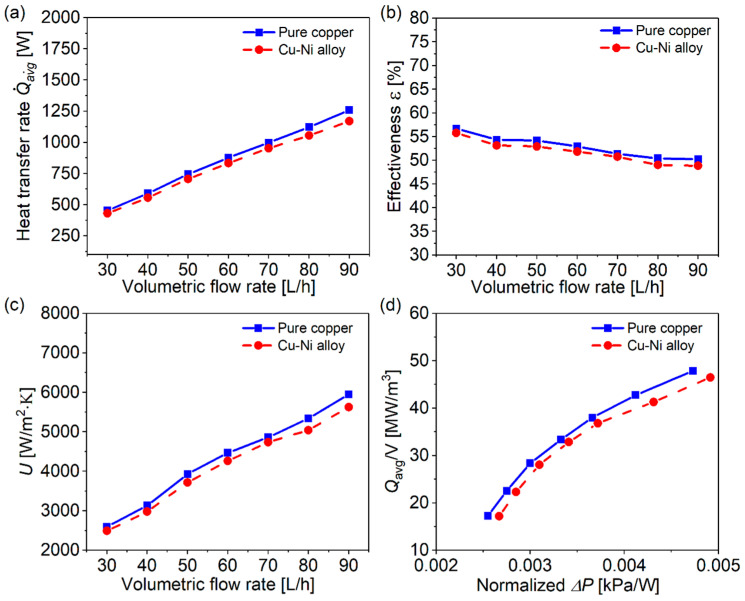
The heat transfer properties of pure Cu and Cu-Ni alloy HEs: (**a**) heat transfer rate; (**b**) effectiveness; (**c**) overall heat transfer coefficient; (**d**) power density per volume versus normalized pressure drop.

To further evaluate their actual performance differences, two distinct heat exchanger configurations were fabricated: one utilizing pure copper (Cu) and another employing a premixed Cu-Ni powder. Figure 11a shows the heat transfer rates of the fabricated Cu and Cu-Ni heat exchangers. Both heat transfer rates increase along with the increment of the volumetric flow rates. This enhanced thermal performance can be attributed to multiple synergistic factors. Firstly, the intensification of turbulent flow characteristics at elevated velocities significantly augments the convective heat transfer coefficient. Secondly, the heat capacity rate of the flow will increase because of the larger temperature difference between the cold and hot water. Both together cause the heat transfer rate to increase as the flow rate rises. The nearly overlapping curves indicate that the two HEs are substitutable in terms of heat transfer rate. The slight difference is due to the difference in thermal conductivity between pure copper and Cu-Ni alloy.

The effectiveness, ε, of fabricated HEs with pure Cu powder and Cu and Ni mixture powder is plotted in Figure 11b. As the flow rate increases from 30 L/h to 90 L/h, the effectiveness of the Cu HE drops from 56.7% to 50.2%, while the value of the Cu-Ni HE drops from 55.8% to 48.9%. Fortunately, the difference in effectiveness between the two is less than 1.3%, which shows that under TPMS structure conditions, the difference in thermal conductivity can almost be compensated for. The observed reduction in thermal effectiveness with increasing flow rate primarily results from the competing effects between the enhanced convective heat transfer coefficients and diminished fluid residence time within the heat exchanger.

The overall heat transfer coefficient (U) values of the fabricated two HEs with pure Cu powder and Cu and Ni mixture powder are shown in Figure 11c. It is found that the value of U increases along with the flow rate increases, and this upward trend exhibits an approximately linear relationship. Furthermore, the U value of Cu HE is slightly higher than that of Cu-Ni HE throughout the whole flow range, and their discrepancy slightly increases at a high flow rate. This indicates that the Cu and Cu-Ni HEs have closer performance at lower flow rates. The increased discrepancy is mainly due to the inability of the boundary layer to be further thinned, consequently increasing the relative contribution of material thermal conductivity to the overall thermal resistance.

Figure 11d presents the relationship between volume-based power density (VBPD) and normalized pressure drop (∆P), where VBPD is defined as the ratio of the total heat transfer rate to the heat exchanger volume. It is found that the VBPD increases with increasing normalized pressure drop. In comparison with the conventional plate–frame and lung-inspired HEs documented in reference [49], the developed Cu and Cu-Ni alloy HEs demonstrate superior performance characteristics. This enhancement can be attributed to the smooth and continuous channels, which allow water to pass through without sudden expansion or contraction. The Cu HE is slightly better than the Cu-Ni HE, and they are good substitutes for each other.

## 4. Conclusions

In this study, we propose using an effective structure design to compensate for the performance loss of materials, thereby achieving the purpose of reusing premixed waste powder in certain non-critical thermal management applications. To validate the idea’s feasibility, Cu and Ni premixed powder obtained from the LPBF in situ alloying process was taken as an example. A comprehensive investigation, including powder characteristics, microstructure, mechanical properties, and heat transfer properties, was then carried out. The following conclusions are obtained:

1. The Cu and Ni premixture exhibits a morphology comparable to that of virgin Cu and Ni powders, and its flowability lies between the two, approaching that of pure Ni.
2. Due to the unalloyed powder, some degree of local inhomogeneities in the composition and texture exists.
3. The compression curve of the Cu-Ni alloy shows a long stress plateau, while the tension curve presents an excellent elongation. The total mechanical properties are close to those of other existing products.
4. The simulated total surface heat fluxes are almost identical to each other.
5. The measured heat transfer rate and effectiveness of the Cu heat exchanger almost coincide with the curves of the Cu-Ni heat exchanger. The maximum difference in heat transfer effectiveness is within 1.3%. The slight variation in heat transfer performance can be attributed to the difference in thermal conductivity between pure copper and the Cu–Ni alloy.

The above investigation shows that the samples printed with the Cu and Ni premixture have good mechanical properties, and the heat transfer performance of the Cu-Ni heat exchanger is similar to that of the Cu heat exchanger. This study demonstrates that effective structure design can partially compensate for the loss of raw material properties, thereby minimizing performance degradation.

## Figures and Tables

**Table 1:** Mesh independence analysis.

Mesh	$T_{hot,out}$ (°C)	Deviation	$T_{cold,out}$ (°C)	Deviation
19.70 M	38.308	−0.39%	36.396	0.63%
33.36 M	38.392	−0.17%	36.294	0.35%
50.91 M	38.457	Baseline	36.167	Baseline
65.51 M	38.460	0.08%	36.172	0.02%

## Data Availability

The data presented in this study are available upon request from the corresponding author.
